# Artificial-intelligence robot umpires in sailing race

**DOI:** 10.3389/fpsyg.2022.979657

**Published:** 2022-11-11

**Authors:** Chien-Hung Wu

**Affiliations:** Department of Marine Recreation, National Penghu University of Science and Technology, Magong, Taiwan

**Keywords:** artificial intelligence, faster-RCNN, federated learning, robot, sailing race

## Abstract

Today, many maritime nations have been promoting boat sports proactively, including sailing races. As sailing races are large-scale regattas that require massive workforces to monitor the game fairly; however, with limited match budgets and labors, Internet of Things (IoT) technology supports monitoring games has become a trend. This article proposes a robot umpire system in sailing races based on Artificial Intelligence (AI) techniques, using drones and AIoT technology to monitor sailing matches. When a large number of sailboats are in a match, and each sail along different routes, drones can monitor the entire game simultaneously. The features of this proposed approach are (1) The system recognizes images by Faster R-CNN, judging whether a sailboat uses a motor to accelerate; (2) The system detects conditions by edge computing; when cheating behaviors happen, it can notify the event holder immediately; (3) Advanced drone route plans can avoid collision incidents; (4) Improve the system recognition by federated learning. This study has implemented an experiment with real drones and installed IoT equipment on the drones for taking videos and recognizing. The experimental result has shown that the proposed approach is feasible and benefits the match's fairness. Additionally, umpires can review the violation details from the videos taken by the drones, supporting evidence for judging.

## 1. Introduction

Sailing is an Olympic sport; it was an official Olympic sport since the first modern Olympic in 1896; yet, due to adverse weather conditions, sailing was canceled in the first event. On the other hand, sailing is a large-scale sport; it requires several umpires taking boats to monitor and maintain fair judgments; consequently, sailing matches need higher labor costs. Despite having many umpires to monitor the game, the time-consuming sport sometimes causes human errors; hence, the addition of robot umpires can enhance the game's fairness. Sailing takes place on the vast sea, the starting point is usually a virtual line, and the competition venue is often a specific area that varies according to the weather conditions. Reaching the first mark usually needs to sail upwind with a zigzag trajectory, which requires massive concentration and consumes heavy physical strength. Sailors control the sailboat moving forward by wind blowing; it is a skill-based and physical strength-assisted sport. A rudder is usually a movable appendage mounted under the boat; moving it can control the direction of the sailboat, which also changes the direction of the sailboat and the wind, as well as the angle between the sail surface and the wind.

Currently, most sailing matches require umpires to take on a boat to monitor and judge the game. With the scale of the game, massive workforces are needed to proceed with the contest; nonetheless, the limited numbers of umpire boats, umpires, and moving ranges, combined with the differences in boat speeds and wind changes, these situations make sailboats scatter in various corners around the sea. Cheating behaviors may occur when umpire boats are not around or when a boat gets stuck in calm waters away from any umpire boats. A robot umpire system is necessary to cope with such a situation and widely monitor the game. A robot umpire system requires intelligence with high mobility; the system can notify the event holder when cheating happens. The equipment in Wang et al. (2007) analyzes dancing reality to identify the movements, and the system will detect the incidental music for better music composition; the film composition through music and dancing can increase the post-production speed. Bass and Pritchett (2008) utilizes a system to detect the environment and the interactions and conflicts between people; the system proceeds the role learning of a judge to analyze the environment and semantics from diverse angles. Moreover, the equipment needs to detect interpersonal body language to check conflicts, developing the system to stand neutral in judging cases. In our research, the drones have been equipped with an AIoT module to monitor sailboats. The AIoT model will proceed with AI detection and check if any sailboats violate the rules; if yes, it will send the results to the judging system, and the umpires can review the violation, improving the fairness of the judgment. The proposed drones also have adaptive cruise control to reduce manual monitoring and enhance the scanning ranges to improve competition fairness.

This research presents an AI robot umpire in sailing races by drones and the AIoT technique to monitor the game. Due to the vast ranges of the game, drones can resolve the territory issue. The proposed drone is designed with an IoT development board and a camera; the AI module in the IoT development board will detect the videos and send the results to the umpires for review. The features of this article are: (1) The equipment uses an IoT development board and a camera to monitor sailboats; (2) The system recognizes images by Faster R-CNN, judging whether a sailboat uses a motor to accelerate; (3) The system detects conditions by edge computing; it can notify the event holder immediately when cheating happens; (4) Advanced drone route plans can avoid collision incidents, and the route plan enables drones to work together and take wide-range videos; (5) Improve the system recognition by federated learning. The proposed system helps umpires to control competition rapidly. Furthermore, the method enables umpires to monitor the entire sailing game from multiple angles. With the Faster R-CNN technique, the system can detect various objects, and the federated learning can update the AI module in the IoT development board and improve the AI recognition. The experiment in a real sailing competition has proved the feasibility of the proposed approach. The main contributions of this article are as follows: (1) Use drones to monitor the competition; (2) Use video to see if there are any violations, and use video evidence to make judgments; (3) Use drones to monitor electronic referees, which will help reduce event personnel costs, (4) Federated Learning improves the recognition of the system.

## 2. Related studies

The suggested AI robot umpire employs drones with cameras to monitor sailing matches and detect violations. Li and Ye (2012) presents transformation estimation to check the moving distances and locations of objects; yet, compared with our research, the proposed method in our research has higher accuracy. The mobile robot that conducts marine autopilot in Jaulin and Bars (2013) uses the robot to do the nautical moving for 100 km; the system can avoid vessel collision and safely arrives at destinations. On the other hand, Corno et al. (2016) has pointed out that sailboat control is challenging; highly non-linear conditions have impacted the boat trajectories and states. Hence, our study has optimized the controller to make sailboat routes more smooth. Meanwhile, Shih (2018) specifically mentions that the data on sporting events are huge. Finding a way to analyze gestures, texts, and referee decisions in videos can help summarize players' conditions and information; these data can support relevant analysis or provide reporters with richer content. In Xie et al. (2018), the system implements license plate recognition by convolutional neural networks. As it takes time and requires more accurate camera equipment to do license plate recognition, the police's hand-held equipment needs to do bias correction regularly to reduce the shaking impact and improve the recognition speed and accuracy. The MD-YOLO architecture used in the research can recognize rotated or oblique license plates, and the experiment result has shown that the recognition rate and speed fulfill the requirement in time.

As mentioned in Jiang et al. (2019), multi-object tracking has always been a research topic in computer vision. This article proposed multi-agent deep reinforcement learning to identify multiple objects in a picture. First, use YOLO v3 to identify objects, and it will track the next picture after finishing the first step; the experimental result has proved the high recognition rate. Viel et al. (2020) resolves the line-up issue of different types of sailboats by following a circular path to maintain a fixed interval between sailboats. There are two challenging issues in sailing: conducting ideal acceleration under unknown wind directions and managing the acceleration directions. The study develops a scheduling strategy to solve the above two problems. On the other hand, as corn harvesters may damage corns while harvesting, Liu and Wang (2019) utilizes YOLO to inspect the corn completeness on the conveyor; the system requires several damaged corn images in training. Kim et al. (2019) operates YOLO v3 to identify objects and compares the method with other approaches, such as DPM, ACF, R-CNN, CompACT, NANO, EB, GB-FRCNN, SA-FRCNN, Faster R-CNN2, HAVD, and UA-DETRAC. The experimental result has shown that the recognition rate of YOLO v3 surpasses the others. Ma and Pang (2019) has mentioned the function of sports medicine, which ensures players' safety and recovery after injuries; the study develops intelligent judgement through medical images, understanding of the condition of the sports injury, and planning the rehabilitation. The sports category algorithm designed in Hsu et al. (2019) presents a wearable sports system and its relevant learning activities. By equipping the devices on athletes' wrists and ankles to collect moving data, the G-sensor on the device will record the signals for analysis. The research can identify the primary sports the user participated in from the CNN algorithm.

In Ghimire and Rawat (2022), it is mentioned that Federated Learning can ensure the safety of the parameters of machine learning in the Internet of Things. In Chen et al. (2022), it is mentioned that deep learning can help detect the points of mechanical failure. In order to improve the recognition degree of deep learning, the parameters of the original deep learning are replaced by Federated Learning, and the security of the parameters is ensured through privacy methods. Chen et al. (2020) proposes an asynchronous learning strategy on the client side and a time-weighted aggregation of the client model on the server. An enhanced federated learning technique is proposed. A framework is proposed in Kumar et al. (2021), which collects a small amount of data from different sources and trains a global deep learning model using blockchain-based federated learning. Then, the model parameters are replaced by Federated Learning to detect the symptoms of patients with COVID-19. Zhang et al. (2021) proposed a novel dynamic fusion-based joint learning method for medical diagnostic image analysis to detect COVID-19 infection. In Islam et al. (2021), artificial intelligence is mainly used for mask detection and blockade detection. In Islam et al. (2021), YoLo V3 is mainly used to identify whether there are people in the blockade. In addition, drones are used to determine whether people are wearing masks. In Islam et al. (2021), the detection range of the camera is wide, so the endurance of drones needs to be considered. In this article, there are mainly multiple drones to be replaced, which can solve the problem of endurance. In Islam et al. (2022), the routing design of drones at each charging station is mainly discussed to serve the power supplement of the drone. In Kim and Moon (2019), drones are mainly used to guide vehicle into the parking lot, and it is guided according to the parking lot route plan.

Xu et al. (2020) has pointed out that road conditions are vital to users while driving because they cannot check multiple messages simultaneously to increase the risk. The article employs YOLO v3 to detect road objects, increasing the controllable road information for drivers. The image processing and 3-dimensional location tracking technology for volleyball matches proposed in He et al. (2020) utilize a particle filter to build a system architecture for volleyball games, effectively improving the tracking accuracy in matches. The medical IoT in Zhang et al. (2020) achieves personnel management, medical equipment management, and automatic drug identification. First, the system collects patients' physiological factors from various sensors to monitor users, such as temperatures and the data from acceleration sensors, helping hospitals analyze patients' exercise conditions. Xia and Fan (2020) offers a healthy IoT technique that benefits the recovery of patients with quadriplegia or stroke. The study receives UE-16B electroencephalography data from a sports-rehabilitation training system and does feature extraction and sorting by MATLAB. In sum, our study identifies image objects by Faster R-CNN and delivers the results to umpires for final judgments, enabling umpires to confirm the violation from the results. Table 1 contains a summary of existing study and its limitations.

**Table 1:** Summary of existing work and its limitations.

**Summary of existing works**	**Limitations**
The work of this article is summarized as follows:	
1. Since the sailing competition venue is	1. The method in this article can only be
large, more referees are required. The	limited to specific sailing events.
method in this article can help reduce	
the problem of the requirement of	
manpower as referees.	
2. This article uses drones to monitor the fairness	2. The method in this article requires multiple
of the competition, and to identify whether the	drones to be alternated, so as to facilitate the
motor is turned on through Faster RCNN.	endurance of drones in the whole event.
3. This article uses Federated Learning to parameterize	3. The method in this article requires photos
the Faster RCNN model to improve the accuracy	of different events to improve the recognition
of image recognition.	accuracy of Faster RCNN.

## 3. The proposed scheme

### 3.1. System model

**Figure 1:**
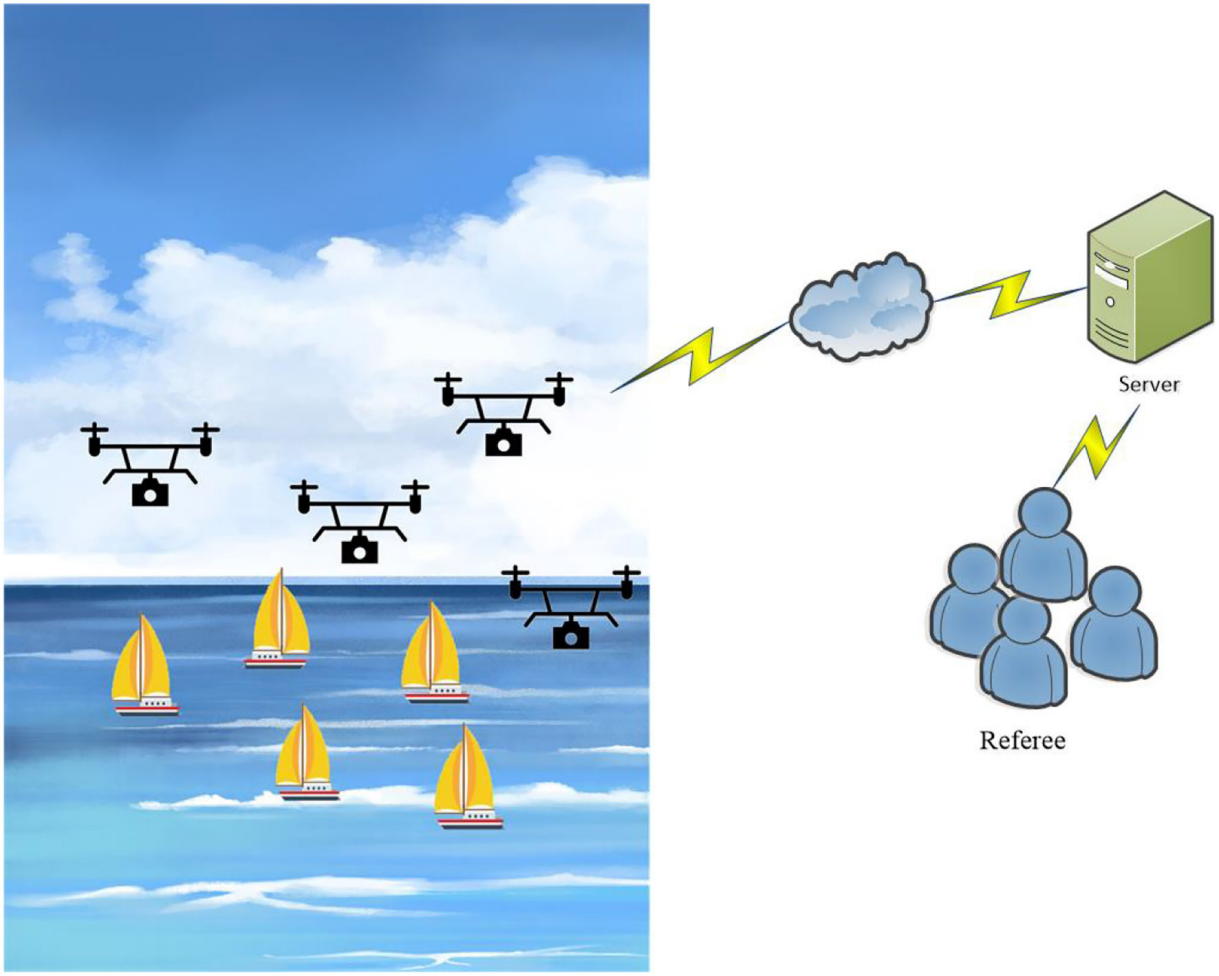
System model.

Figure 1 demonstrates the system model; a drone has installed an IoT development board that consists of the below functions: (1) Sailboat object detection by Faster R-CNN; (2) To check if a boat uses a motor to accelerate and violate the rule; (3) The 5G Network will identify the violation, send the video to the server, and notify umpires for confirmation. The drones employ a cruise mode that uses graph coloring to set the route and avoid having drones in the same area; assume that drones cruise from the opposite direction corresponding to the graph, benefiting the drones to conduct large-scale monitoring. Additionally, the IoT development board will proceed with intelligent recognition and edge computing. Edge computing can reduce communication and calculation costs; the videos can obtain an outcome from the calculation on the IoT development board, avoiding delivering massive videos to the server to occupy storage and calculation workload. Moreover, to improve the video's recognition range, the model uses federated learning to update the Faster R-CNN module parameters in each drone; the modules do not need to transmit videos to the server for training but merely send the module parameters for optimal solution calculation. Apart from enhancing the module's recognition rate, federated learning can also protect the privacy of relevant videos. The experimental result has proved that the method has boosted fairness by monitoring sailing races; the recognition rate of the experiment is decent, which significantly improves the game's fairness.

### 3.2. Faster R-CNN

This article utilizes Faster R-CNN to conduct object recognition in pictures, cutting the recognized boat objects into pieces to check if any of them run a motor to accelerate. The flow chart of Faster R-CNN is illustrated in Figure 1. Fast R-CNN treats the entire image and multiple region proposals as the input; next, process the whole image through the convolution and pooling layers to produce convolution eigenvalues. For each region proposal, the RoI pooling layer will extract a fixed-length eigenvector from eigenvalues and send each eigenvector into a series of the fully connected layer. Finally, divide the results into two same-level output layers; one outputs K types and one Softmax prediction as to the background type, and the other outputs four real values from those K types. The four values of each group mean the position correction of the type's check box. A Fast R-CNN network has two output layers; one outputs the discrete probability distribution of the *K*+1 types, and the other outputs the reset of the bounding box regression. The formula of the loss function is:


(1) $$\begin{array}{r} L\left(p,u,t^{u},v\right)=L_{cls}\left(p,u\right)+\lambda \left[u\geq 1\right]L_{loc}\left(t^{u},v\right) \end{array}$$

Function (1) is the loss function for regression, *t*^*u*^ represents the prediction result, *u* means types, and *v* is the real result.

The second part is the loss regression, where *V* is the ground truth, which adopts *smooth*_*L*1_ loss toward the regression of the checking box. The calculation is as follows:


(2) $$\begin{array}{r} L_{loc}\left(t^{u},v\right)=\underset{i\in \left\{x,y,w,h\right\}}{\sum }smooth_{L_{1}}\left(t_{i}^{u}-v_{i}\right) \end{array}$$

Regarding image sorting, most of the processing time is consumed in the convolution layer, and the system will spend less time in the fully connected layer. However, target detection algorithms usually need to handle massive ROI, which spends more time on the fully connected layer. Therefore, Truncated SVD can easily accelerate on the large fully connected layer; the system can rapidly highlight the target objects in images and further detect if the object uses a motor.

### 3.3. Federated learning

Federated Learning mainly uses the Public Key Infrastructure architecture for private communication, in which the Hash function is used for message integrity confirmation. First, each device has a Public Key (*PK*_*i*_), Private Key (*PR*_*i*_), and a certificate (*Cer*_*i*_). Each device will use the public key of the server to encrypt the parameters of the device and uses the Hash function to calculate the message so that the server can confirm the integrity of the message, and the server will optimize the parameter calculation after collecting the information of each device. Federated learning can sort information based on the types of information, primarily are (1) Horizontal learning; (2) Vertical learning; (3) Federated transfer learning. Our study employs horizontal learning to train the parameters, and the process is as follows:

1. When initiating each IoT development board, the system will update parameters with the server.
2. Each IoT development board will encrypt the parameters and send the data to the server; the server will calculate the optimal solution by gradient descent when collecting all of the parameters.
3. The server will then transmit the optimal solution to each IoT development board.
4. Each IoT development board will update the model parameters separately.

During the federated learning training, there are server datasets, $\left\{x_{i}^{A}\right\},i\in D_{A}$, each IoT development board parameter dataset, $\left\{x_{i}^{B},y_{i}^{B}\right\},i\in D_{B}$, and the initialization parameter model sets, θ_*A*_ and θ_*B*_; the objective function is:


(3) $$\begin{array}{r} \underset{\theta_{A}\theta_{B}}{\min}||\theta_{A}x_{i}^{A}+\theta_{A}x_{i}^{A}-y_{i}|{|}^{2}+\frac{\lambda }{2}\left(||\theta_{A}|{|}^{2}+||\theta_{B}|{|}^{2}\right) \end{array}$$

Set $u_{i}^{A}=\theta_{A}x_{i}^{A},u_{i}^{B}=\theta_{A}x_{i}^{B}$, where [[]] is the encryption symbol; the encrypted objective function is:


(4) $$\left[\left[L\right]\right]=\left[\left[\sum_{i}{\left(u_{i}^{A}+u_{i}^{B}-y_{i}\right)}^{2}+\frac{\lambda }{2}\left({\left\|\theta_{A}\right\|}^{2}+{\left\|\theta_{B}\right\|}^{2}\right)\right]\right]$$

Next, using gradient descent to calculate the objective function, the parameters of the server and IoT development boards are:


(5) $$\left[\left[\frac{\partial L}{\partial \theta_{A}}\right]\right]=\sum_{i}\left[\left[d_{i}\right]\right]x_{i}^{A}+\left[\left[\lambda \theta_{A}\right]\right]$$

(6) $$\left[\left[\frac{\partial L}{\partial \theta_{B}}\right]\right]=\sum_{i}\left[\left[d_{i}\right]\right]x_{i}^{B}+\left[\left[\lambda \theta_{B}\right]\right]$$

Finally, the server will update the parameters $\left(\left[\left[\frac{\partial L}{\partial \theta_{B}}\right]\right]\right)$ and send the encrypted data to IoT development boards, and the IoT will update the module after receiving the parameters.

### 3.4. Routh planning for drones

**Figure 2:**
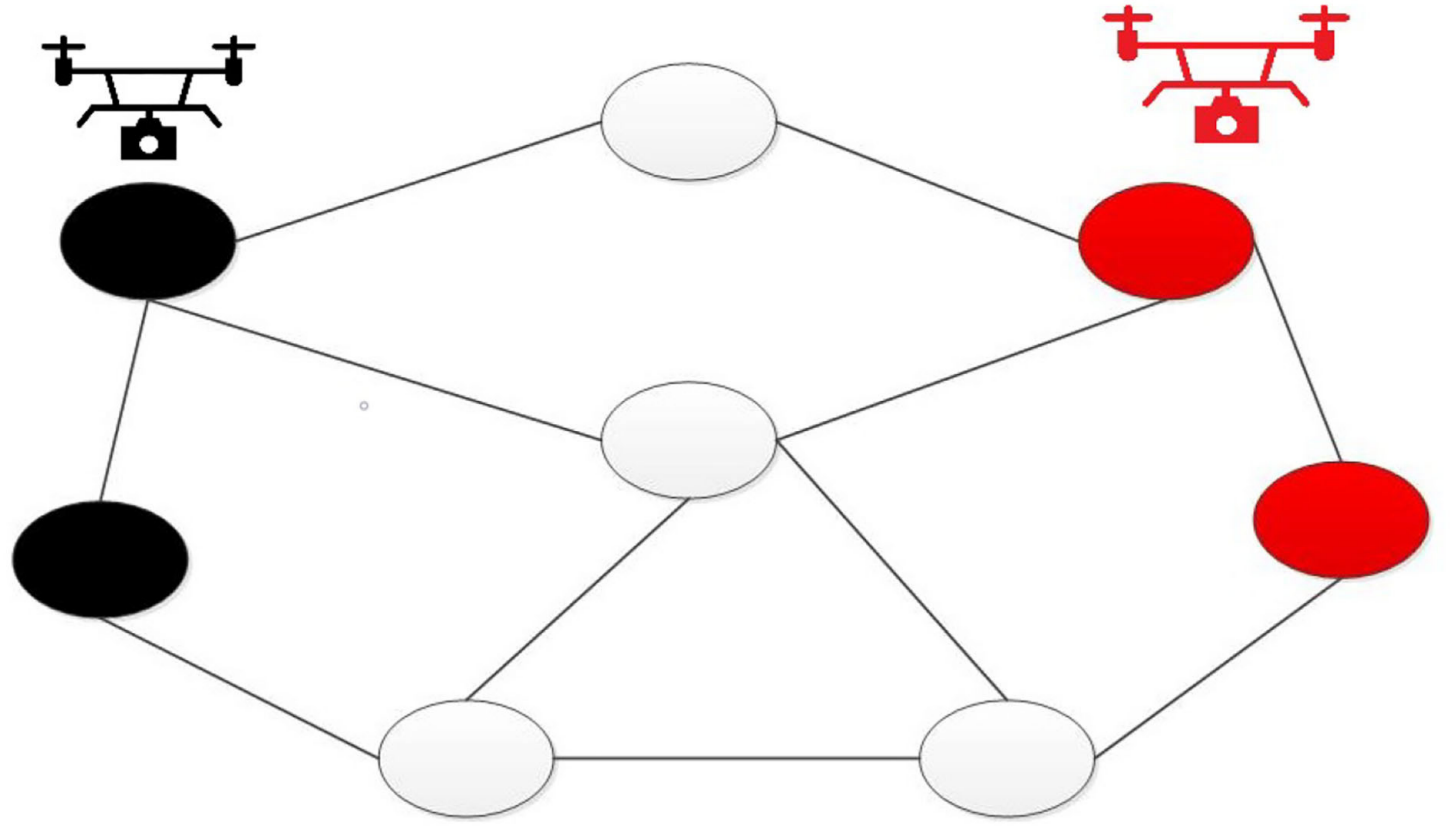
A route plan for drones.

This article utilizes a graph coloring problem to plan routes for drones. Because sailing moves in a large range, it requires several drones to monitor the match. We set n numbers of vertexes (*V*), and the location of vertexes can be identified in a GPS. Thus, shape the undirected graph *G* = (*V, E*), where *E* is the side set; set there are m numbers of drones, each of them will be at a different vertex. The vertex coloring theory will separate the drones on different vertex, *m* numbers of drones will have *K* numbers of colors on the map as adjacent vertex should mark with different colors. Therefore, the drones will cruise from different vertexes to achieve comprehensive monitoring. In other words, because each drone locates differently, the situation of repeat monitoring will not happen. Figure 2 is a route plan for drones. Once a drone takes a photo showing that a sailboat runs a motor, the system will send an alarm to the server, and the server will notify umpires to review the violation and judge the situation. This article will set up the drone charging station at the competition venue. When the drone battery reaches low power, it will return to the charging station, replace the battery and return to the competition venue to continue monitoring operation.

## 4. Experiment results

**Figure 3:**
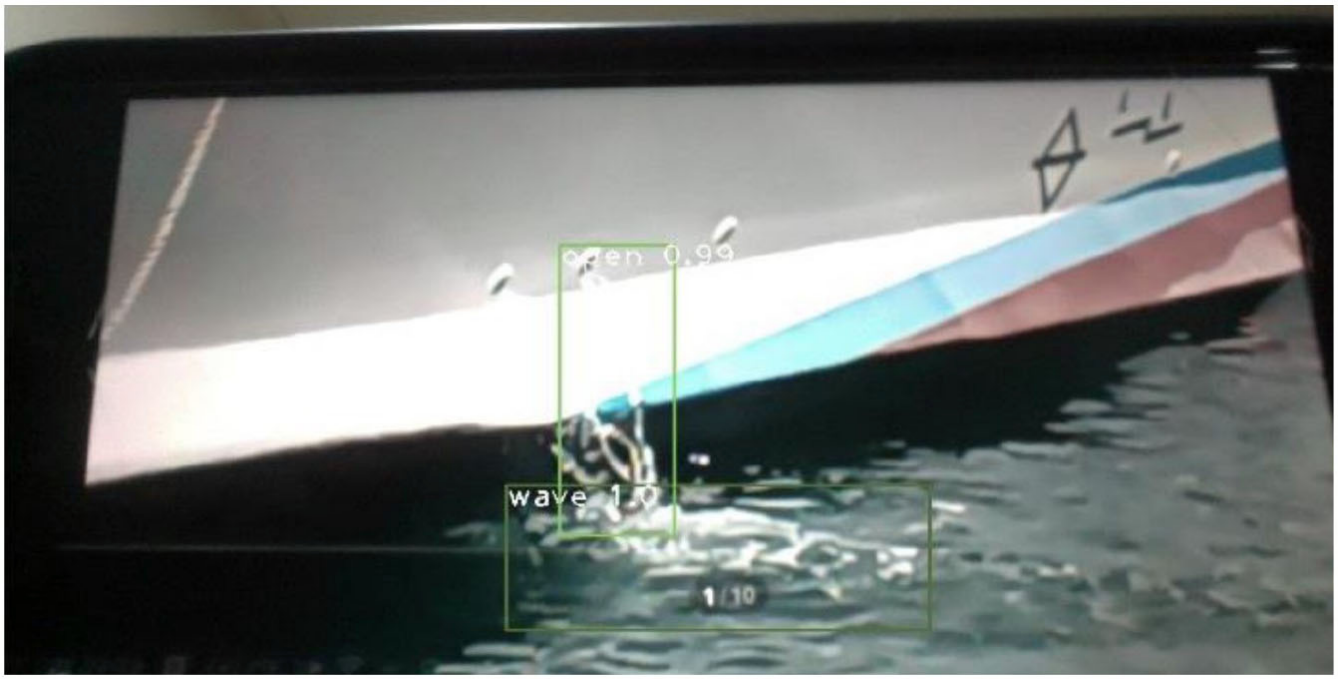
Monitoring if any sailboat violates the rule to run a motor.

**Figure 4:**
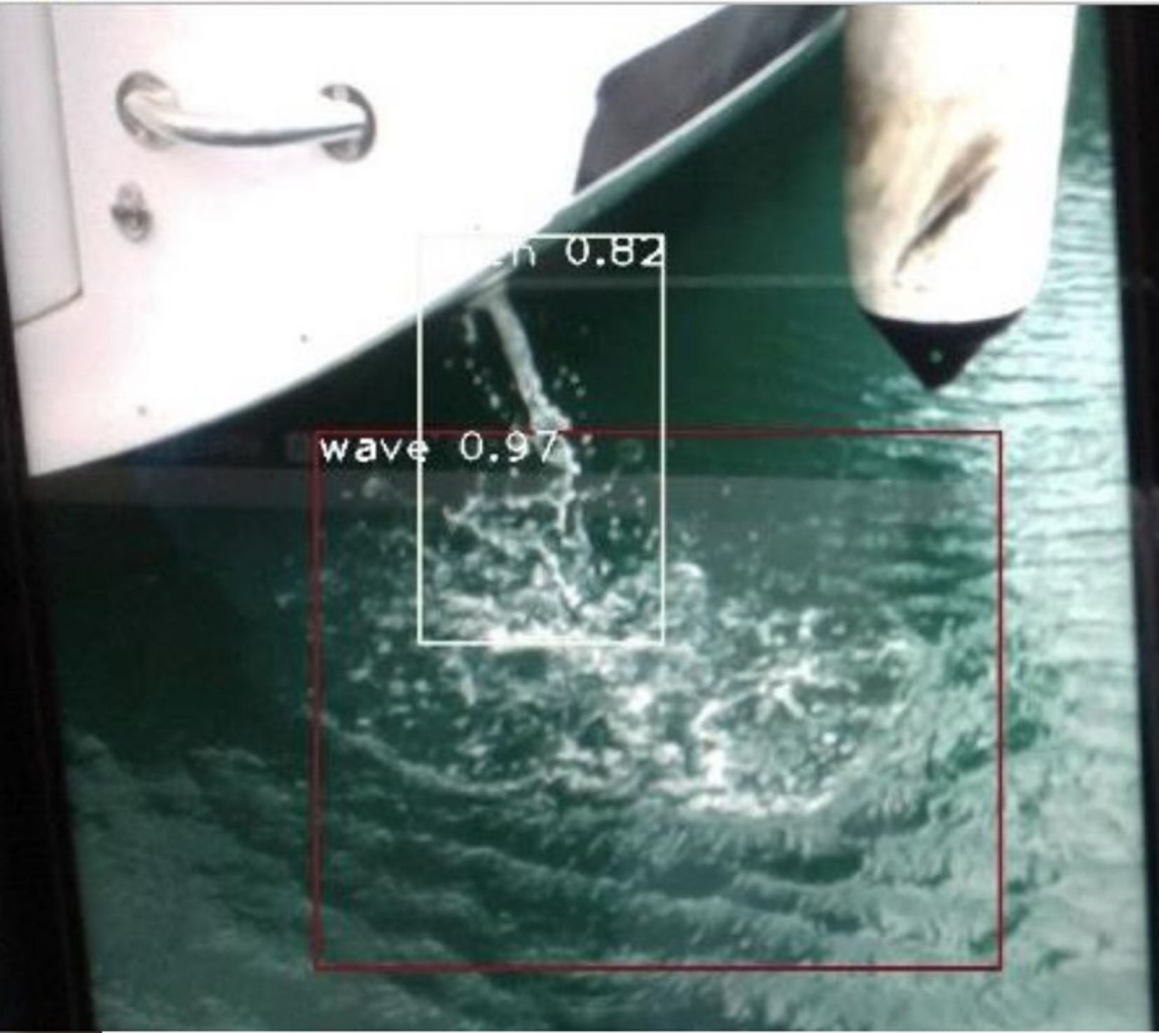
The flow chart of the Raspberry Pi sending the results to the umpires.

**Figure 5:**
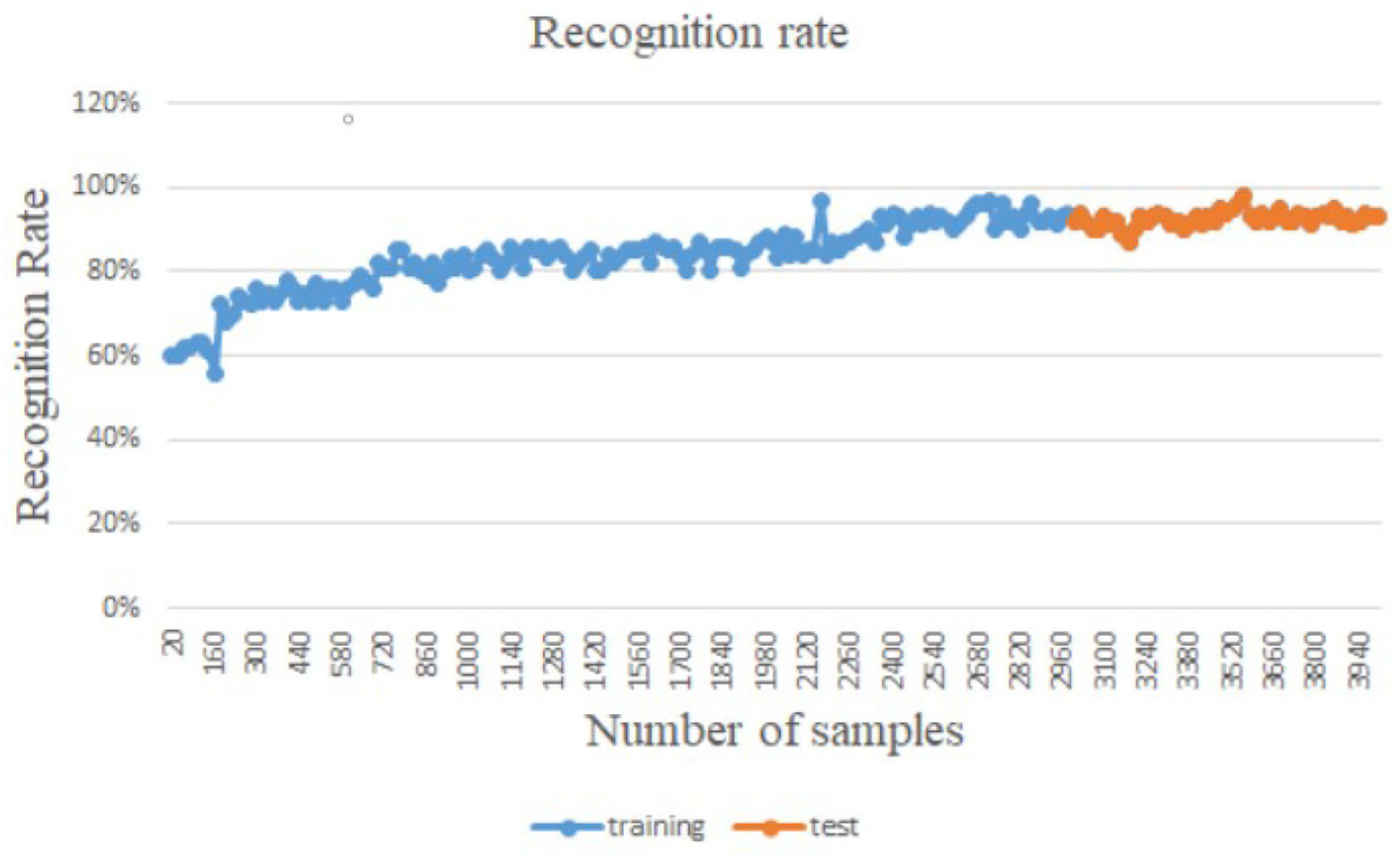
Faster R-CNN detection results.

**Figure 6:**
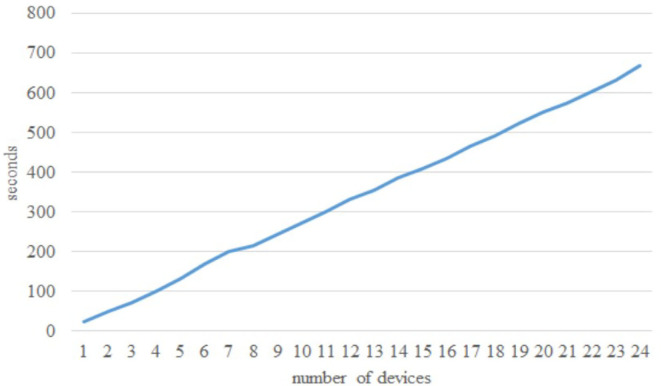
Federated learning execution effectiveness.

**Figure 7:**
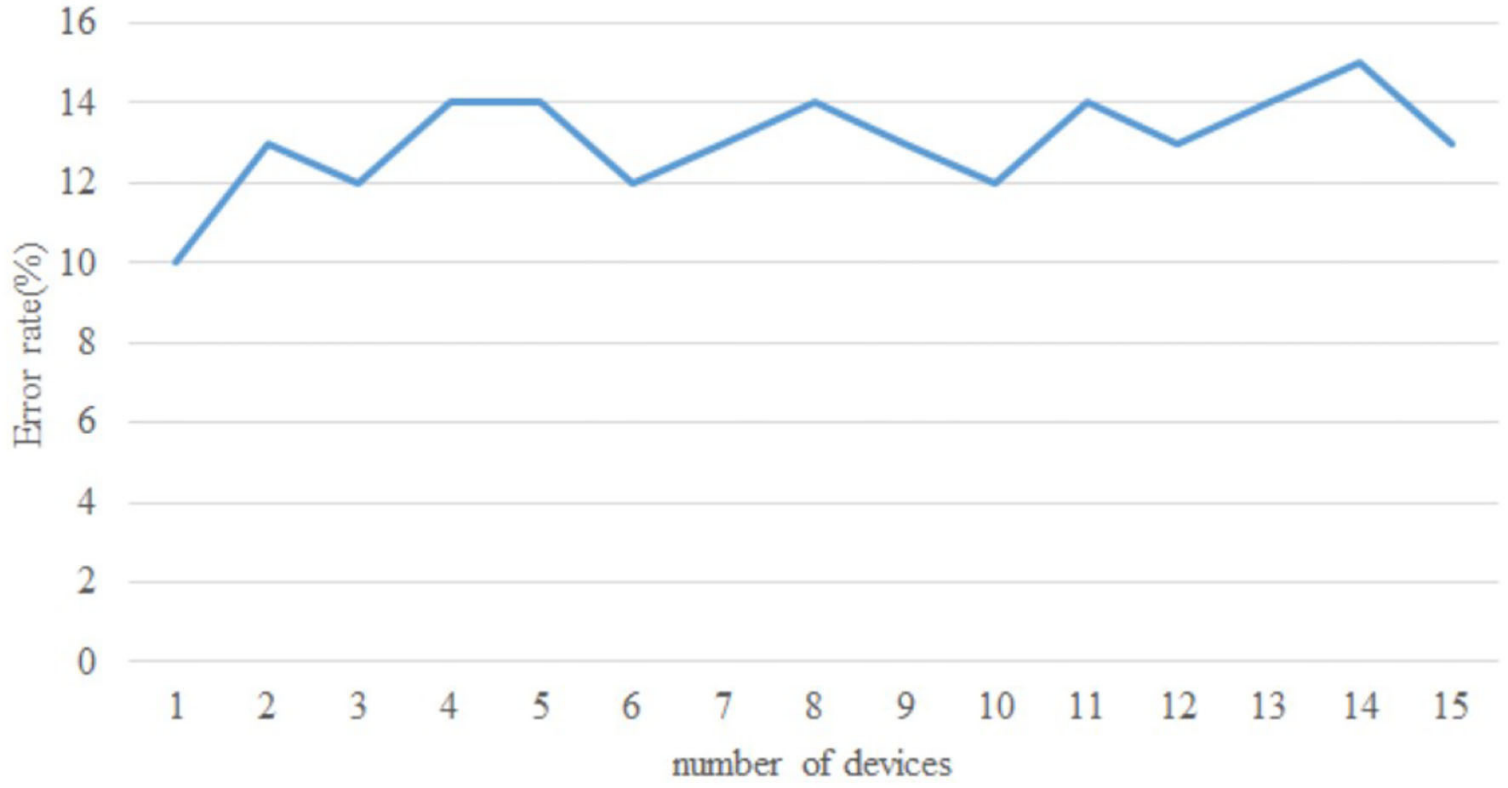
Route planning error rate.

**Figure 8:**
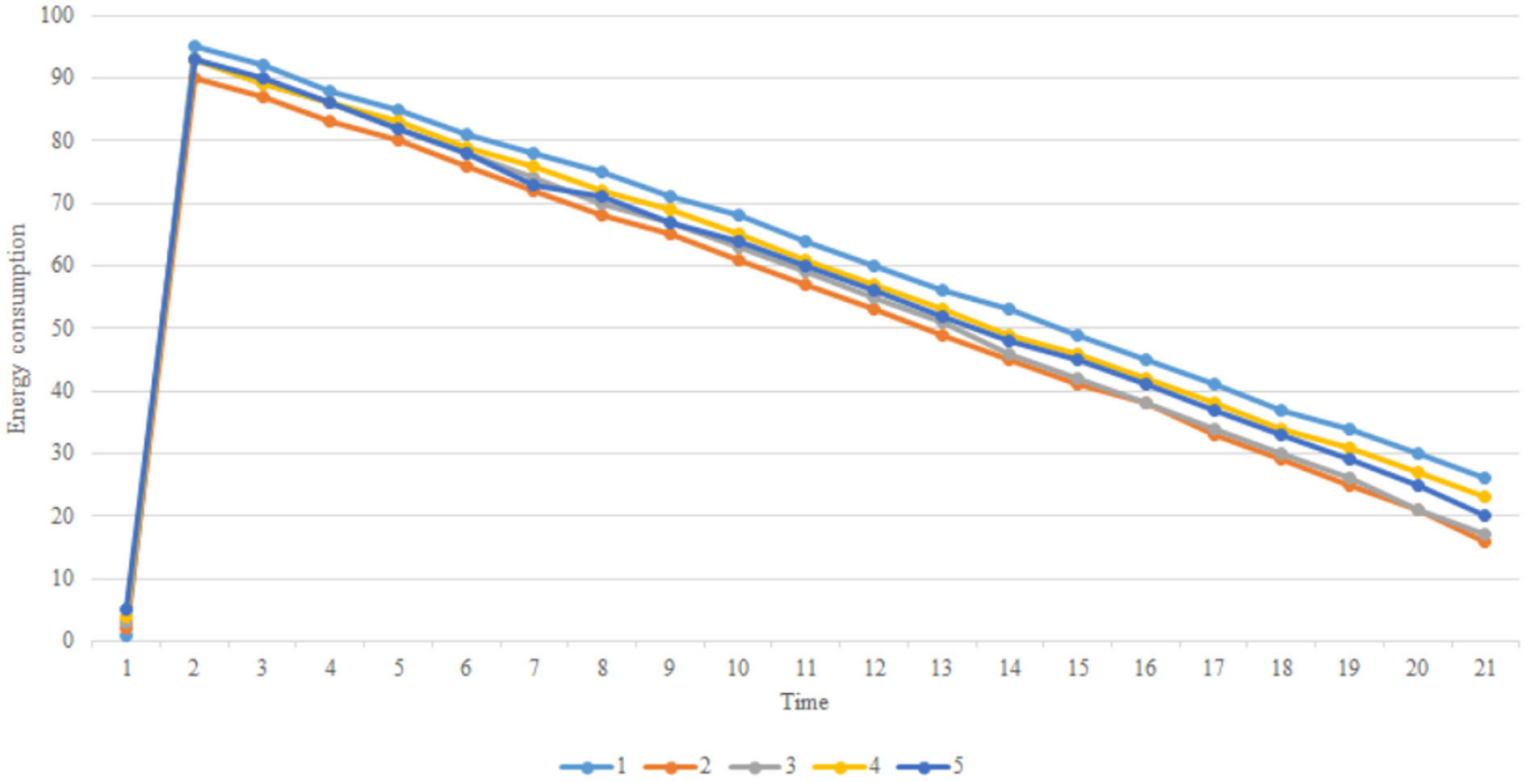
Aerial camera energy consumption.

The article develops an AI robot-umpire system in sailing, using drones to monitor the matches, and the IoT development board of the system will deliver the detection results to the server when noticing violations. The hardware and software equipment is listed in Table 2. Our system uses Python to develop the model and monitor games by Raspberry Pi with a camera. The faster R-CNN technique runs calculations in Raspberry Pi; furthermore, Raspberry Pi can send the results to the server through the 5G Network. Additionally, Raspberry Pi will deliver parameters to the server for training; when the server obtains an optimal solution, it will send the results back to Raspberry Pi for updating the model. As shown in Figure 3, when Faster R-CNN detects any sailboat runs a motor by discovering the motor pumps water out. The Raspberry Pi will send the result to the umpires to review and judge the situation, which is shown in Figure 4. Figure 5 reveals that the recognition rate of Faster R-CNN is higher than 90%. The experimental result has proved that the suggested approach is feasible, and the system can detect whether a sailboat violates the rules. Figure 6 is mainly the execution performance of Federated Learning. This article mainly analyzes the photos of each event, so the calculation performance can be updated on the same day. Figure 7 is the main path planning. The drone has the GPS location of the path, so there are fewer cases of path deviation. Figure 8 is the analysis of the drone energy consumption.

**Table 2:** The list of hardware and software equipment.

**Hardware development tools**	**Software development tools**
Raspberry Pi	Windows 10
Drone	Python
5G Network	Faster-RCNN
Video camera	
GPS	
Server	

## 5. Conclusion

With the popularity of sea sports, the diversity has also increased, especially in boat competitions. However, the large-scale venue also makes it challenging for human monitoring; for example, the limited number of umpires cannot notice boat violations in detail at all times. This research constructs an AI robot-umpire system in sailing races; the drones can conduct comprehensive monitoring and enhance the game's fairness. Using the Faster R-CNN technique, the system fairly monitors the game; the experimental result has proved the feasibility of checking violations and sending judgments to the umpires for the final ruling. In the future, this suggested approach can further monitor moving trajectories, detecting which boat reaches the mark first and sending the result back to the umpires. Furthermore, drones can monitor the entire match and obtain the game conditions from diverse angles, the development will significantly improve the fairness of the game.

## Data availability statement

The raw data supporting the conclusions of this article will be made available by the authors, without undue reservation.

## Author contributions

CW: study conception and design, analysis and results, draft the manuscript preparation, reviewed the results, and approved the final version of the manuscript.

## Conflict of interest

The author declares that the research was conducted in the absence of any commercial or financial relationships that could be construed as a potential conflict of interest.

## Publisher's note

All claims expressed in this article are solely those of the authors and do not necessarily represent those of their affiliated organizations, or those of the publisher, the editors and the reviewers. Any product that may be evaluated in this article, or claim that may be made by its manufacturer, is not guaranteed or endorsed by the publisher.
